# Prospective payment systems and discretionary coding—Evidence from English mental health providers

**DOI:** 10.1002/hec.3851

**Published:** 2018-12-27

**Authors:** Giuseppe Moscelli, Rowena Jacobs, Nils Gutacker, Maria Jose Aragón, Martin Chalkley, Anne Mason, Jan Böhnke

**Affiliations:** ^1^ School of Economics University of Surrey Guildford UK; ^2^ Centre for Health Economics University of York York UK; ^3^ Department of Health Sciences University of York York UK; ^4^ Dundee Centre for Health and Related Research, School of Nursing and Health Sciences University of Dundee Dundee UK

**Keywords:** classification, discretionary behaviour, episodic payment, hospitals, mental health, mixed‐effects models

## Abstract

Reimbursement of English mental health hospitals is moving away from block contracts and towards activity and outcome‐based payments. Under the new model, patients are categorised into 20 groups with similar levels of need, called *clusters*, to which prices may be assigned prospectively. Clinicians, who make clustering decisions, have substantial discretion and can, in principle, directly influence the level of reimbursement the hospital receives. This may create incentives for upcoding. Clinicians are supported in their allocation decision by a clinical clustering algorithm, the Mental Health Clustering Tool, which provides an external reference against which clustering behaviour can be benchmarked. The aims of this study are to investigate the degree of mismatch between predicted and actual clustering and to test whether there are systematic differences amongst providers in their clustering behaviour. We use administrative data for all mental health patients in England who were clustered for the first time during the financial year 2014/15 and estimate multinomial multilevel models of over, under, or matching clustering. Results suggest that hospitals vary systematically in their probability of mismatch but this variation is not consistently associated with observed hospital characteristics.

## 1 INTRODUCTION

Most mental health services in England are provided as a part of the publicly funded National Health Service (NHS) and are free at the point of delivery to patients. These services have primarily been funded through fixed budgets independent of the actual services delivered (*block contracts*) agreed between purchasers (*commissioners*) and providers of care (Mason, Goddard, Myers, & Verzulli, 2011). These arrangements have typically been negotiated on the basis of historical expenditure. From April 2017, mental health (MH) providers were to be remunerated through one of two approaches (NHS England, 2016; NHS Improvement, 2017): (a) a *capitation* payment model, which is a per‐person risk adjusted sum to cover a range of care for the population across a number of different settings or (b) an *episodic payment* model, which rewards providers according to the number and type of patients they treat, and sometimes the quality of care they provide, similar to the prospective payment system (PPS) used to fund acute hospital care in England and other countries (Khan, Nowak, & NHS England, 2014; O'Reilly et al., 2012; Sood, Buntin, & Escarce, 2008). In both payment approaches, prices for mental health care (either per patient or per treatment) are set locally.

In this article, we focus on the episodic payment system. In this system, patients are categorised into one of 20 *clusters* according to need, and these clusters are grouped into one of three *superclasses* (nonpsychotic, psychotic, organic) depending on the prevalent profile and MH disorder of the patient (see Table 1). 1There are 20 clusters; 0–21, with 9 being blank, and 0 a variance cluster, which is used if no other suitable cluster can be found, the use of which should reduce over time. We consider 19 clusters (0–21, excluding 0 and 9). Under the episodic payment approach, each cluster will attract a fixed daily price, which is different for inpatient (*admitted*) and outpatient (*nonadmitted*) care. Table 1 provides the average cost for an episode of care by cluster across all hospitals. Clusters also define the relevant period of care, and the system requires patients to be reviewed and assigned to clusters according to those periods. The clusters are mutually exclusive meaning that a patient should only be assigned to one cluster at any given time.

**Table 1:** Superclasses and clusters

Superclass	Cluster number	Cluster label	Average cost per episode^a^ (in £)	Order by average cost
Nonpsychotic	1	Common mental health problems (low severity)	1,293.92	1
	2	Common mental health problems	1,539.66	2
	3	Nonpsychotic (moderate severity)	1,741.27	3
	4	Nonpsychotic (severe)	2,232.43	4
	5	Nonpsychotic (very severe)	3,201.27	6
	6	Nonpsychotic disorders of overvalued Ideas	2,870.06	5
	7	Enduring nonpsychotic disorders (high disability)	3,226.87	7
	8	Nonpsychotic chaotic and challenging disorders	4,218.92	8
N/A	9	Blank cluster		
Psychotic	10	First episode in psychosis	5,541.50	3
	11	Ongoing recurrent psychosis (low symptoms)	3,083.34	1
	12	Ongoing or recurrent psychosis (high disability)	4,741.86	2
	13	Ongoing or recurrent psychosis (high symptom and disability)	7,709.77	6
	14	Psychotic crisis	9,088.57	7
	15	Severe psychotic depression	6,859.16	5
	16	Dual diagnosis (substance abuse and mental illness)	6,132.45	4
	17	Psychosis and affective disorder difficult to engage	9,353.91	8
Organic	18	Cognitive impairment (low need)	974.58	1
	19	Cognitive impairment or dementia (moderate need)	1,728.65	2
	20	Cognitive impairment or dementia (high need)	3,790.52	3
	21	Cognitive impairment or dementia (high physical need or engagement)	5,291.61	4

*Note*. Cluster 9 is not used. Cluster 0 (not shown) is a variance cluster to which allocations can be made if no other suitable cluster can be found, but which should be used less over time.

a The average cost per episode is computed across all hospitals in year 2013/14, as specified in Equation (1), Section 3.

Patients are assigned to a cluster by a clinician or clinical team, who can be assisted in their assignment process (known as “clustering”) by an algorithm, called the Mental Health Clustering Tool (MHCT). The paper‐based MHCT, which has been recommended for use since 2013 (Monitor and NHS England, 2013a, 2013b), consists of 18 items and combines information from the 13 items of the Health of the Nation Outcomes Scales (HoNOS; Wing, Curtis, & Beevor, 1994), a routine outcome measure used in mental health services, and the five items of the Summary Assessment of Risk and Need (SARN) instrument (Self, Painter, & Davis, 2008; Self, Rigby, Leggett, & Paxton, 2008), which assesses need and risk on both a current and historical basis (see Online Appendix Table A.I). A computerised version of the MHCT algorithm has been developed to support clinicians and provides a probability of a patient being assigned to a particular cluster. The MHCT has been designed “to ensure consistency of clustering and to improve the overall accuracy of cluster allocation” (McKenna, 2012). 2The documentation of the algorithm can be found here: http://webarchive.nationalarchives.gov.uk/20130507000015/https:/www.gov.uk/government/publications/mental‐health‐pbr‐road‐test‐package‐for‐2013‐14. A clinician is, however, able to override the algorithm allocation and the ultimate classification is based on clinical judgement.

The reimbursement of mental health care will be based on the patients' categorisation into clusters. In particular, the proposed episodic payment approach links a provider's payment to the volume and type of mental health care *activity*, independently of how much treatment any individual patient receives or how that treatment is delivered and is thus a form of PPS. The potential advantages and risks of PPS have been discussed extensively in the literature (Charlesworth, Davies, & Dixon, 2012; Jacobs, 2014). One key risk is the potential of *upcoding* in which providers assign patients to categories that maximise payment but do not appropriately reflect patients' needs (Dafny, 2005; O'Reilly et al., 2012). In mental health services, upcoding is possible because clustering is performed by members of the clinical team rather than by clinical coders. With only 20 clusters, clinical teams may, to varying degrees, be aware of the relative monetary value attached to each. The use of the computerised version of the MHCT is not mandatory, 3Although anecdotally, it is believed to be used in most services, there is no information regarding how widespread the use of this algorithm is. and its suggested cluster allocation can be manually overridden.

Although the allocation of patients to clusters other than that recommended by the MHCT algorithm could represent an appropriate clinical decision, it could also, intentionally or unintentionally, benefit hospitals financially (Jacobs, 2014). Some random variation in clustering is expected because care needs varying across patients and not all risk factors are observable or recorded. This has little effect on providers' reimbursement because the *expected* payment for a given (latent) patient type is unaffected. Conversely, any systematic coding differences across providers of care would raise concerns because it potentially results in an inappropriate allocation of financial resources. Systematic differences may arise because of differences in unmeasured case mix across providers 4For example, the clusters' definitions may lead to a systematically misclassification of patients in favour of some providers over others due to unobserved patients' characteristics. or because providers engage in discretionary coding to their advantage. Either mechanism calls into question the appropriateness of reimbursing MH providers based on clusters.

This work is the first to assess whether providers differ systematically in their coding behaviour and whether this is associated with their observable characteristics, such as the average cost of care. In doing so, we provide the first comprehensive assessment of the coding behaviour of all NHS MH providers in England by exploiting a large, national patient‐level data set. We test whether the clustering process is subject to upcoding by MH providers, defined as positive discrepancies between patients' assignment to cluster by clinicians and the cluster allocation suggested by the MHCT algorithm. Importantly, having an external standard, the MHCT, against which observed coding can be compared, is a unique feature of our study and sets us apart from the existing literature on discretionary coding in PPS.

## 2 RELATED LITERATURE

Concern that a payment mechanism that relies on the classification of patients by clinicians might be subject to distortion or manipulation first arose with the adoption of PPS by the U.S. Medicare in 1983, although the potential for distortion had been previously recognised (Simborg, 1981). Under Medicare PPS, patients are reimbursed according to the diagnosis‐related group (DRG) that they are allocated to and hospitals are perceived to have discretion over the allocation (Ellis & McGuire, 1986).

The nature and manifestation of that discretion has been subject to considerable debate and research. At one extreme, the falsification of records or deliberate distortion of evidence constitutes fraud (Jesilow, 2005), and such a possibility has given rise to an active debate on how hospital payment systems might need to be policed and audited (Kuhn & Siciliani, 2008). Less extreme is the possibility that treatment decisions and care pathways might be influenced by the desire to allocate a patient to a better paid (or better resourced) DRG (Rosenberg & Browne, 2011).

The empirical investigation of these phenomena was driven by the observation of increasing costs arising from more complex and costly bundles of DRGs being observed overtime: A phenomenon referred to as *DRG creep* (Simborg, 1981). If DRG creep is not a consequence of patients getting sicker, or of more sophisticated but appropriate treatments being used, it is conjectured likely to be a manifestation of hospitals *upcoding*—deliberately increasing the complexity of the procedures that they undertake—and there is now credible evidence that this exists in practice, both for the U.S. and for other health care systems that have adopted DRG mechanisms (Silverman & Skinner, 2004; Steinbusch, Oostenbrink, Zuurbier, & Schaepkens, 2007).

Besides indicating that the risks of upcoding and other forms of manipulation 5The use of *downcoding* to reduce the risk of audit has also been considered http://www.racmonitor.com/rac‐enews/2150‐medicare‐audits‐drg‐downcoding‐in‐hospitals‐algorithms‐substituting‐for‐medical‐judgment‐part‐i.html
 are real, the literature points to some potentially important determinants. First, because manipulation may be motivated by financial returns, one hypothesis is that for‐profit health care providers may be more inclined to upcode. Both theoretical and empirical support for this is, however, mixed. In regard to theory, not‐for‐profit providers may still desire to produce a financial surplus in order to further their own goals. In practice, the relationship between managers and clinicians, rather than the overarching goals of the providing organisation, appears to be a more important driver for upcoding (Silverman & Skinner, 2004). Second, the design of any DRG system would seem to be important in limiting or facilitating manipulation. Systems that rely on objective, medically meaningful criteria are inherently more resistant to manipulation, whereas the more complex a system becomes and the greater the proliferation of DRGs, the greater the risk (Steinbusch et al., 2007).

The phenomena of DRG creep and upcoding have predominantly been considered in relation to acute hospital physical health services, following the broad adoption of the PPS systems for hospital services, which started in the United States and spread extensively to Europe (Ellis & McGuire, 1986; Steinbusch et al., 2007). Translating the insights into the mental health care context considered in this paper poses challenges. Relative to most acute care payment systems, which have hundreds to thousands of DRGs, 6For example, there are 747 DRGs in the U.S. Medicare (https://www.cms.gov/icd10manual/fullcode_cms/P0368.html), while 538 DRGs are used for hospital treatment reimbursement in Italy (Lanzarini et al., 2014); in the United Kingdom, there are over 1,400 Health care Resource Groups (http://content.digital.nhs.uk/hrg4)—the U.K. equivalent of DRGs. the MH clustering system, we consider is simple and limited. However, the *criteria* upon which clustering is undertaken, seem *a priori* to be subject to clinician discretion, which in turn may be an inherent characteristic of care for mental illness (Goldman & Grob, 2006; Bellows & Halpin, 2008). Hence, our analysis of the extent of provider discretion within this emerging system is of importance to health policy makers in framing the development of mental health care payment systems.

## 3 DATA

The analysis uses administrative data from the Mental Health Services Data Set, which covers 53 English NHS MH hospital trusts (the *providers*). For each patient, we obtain the observed cluster allocation as well as a rich set of individual‐level variables, including patients' gender, age (coded in age bands), marital status (single, married, separated, divorced, undisclosed), ethnicity (White, Black, Asian, other), and approximate level of deprivation at small area level (in quintiles). Patients' residence is reported at small area level (the Lower Layer Super Output Area). Each small area includes approximately 1,500 inhabitants and is designed to be homogeneous with respect to tenure and accommodation type. We use Lower Layer Super Output Areas defined according to 2001 Census boundaries by the English Office for National Statistics. 7There were 32,482 LSOAs in England according to this definition. We link this geographic identifier to the 2010 Index of Multiple Deprivation (IMD) to approximate deprivation levels at small‐area level (McLennan, Barnes, Noble, & Dibben, 2011; Noble, Wright, Smith, & Dibben, 2006). Information on patient severity is provided by the ratings on the HoNOS and SARN instruments.

We restrict our analysis to patients who had not been clustered between April 1, 2011, and March 31, 2014, and who received treatment between April 1, 2014, and March 31, 2015. These dates are determined by the financial years that are used in recording data. Patients who have been clustered during a previous care episode may be at risk of having their cluster allocation carried forward without detailed review; that is, any subsequent clustering may not be independent of previous decisions. We therefore exclude all patients who have received treatment as recorded in Mental Health Services Data Set data in the previous 3 years (266,100 patients), and any subsequent clustering after the first episode between April 1, 2014, and March 31, 2015 (18,526 patients). 8Note that patients in our sample may have been treated in primary care by their general practitioner, where clustering is not used, or by mental health hospitals prior to the financial year April 1, 2011, to March 31, 2012, which predates the introduction of clusters. To ascertain the persistence of clustering assignment for the same patients over time, we estimate the polychoric correlations (Kolenikov & Angeles, 2004; Olsson, 1979) between current and past clusters, separately by superclass.

For each patient, we observe the actual cluster allocation given for their first episode and further calculate the most likely cluster using the computerised MHCT algorithm (http://www.cppconsortium.nhs.uk/algorithm/). The algorithm requires the user to choose a superclass (nonpsychotic [clusters 1–8], psychotic [10–17] or organic [18–21]) 9The superclass selected for the purpose of the MHCT was not recorded for 13% of observations in our sample. We used the superclass implied by the actual, observed cluster allocation on the grounds that clinicians will generally be able to ascertain the superclass with a high degree of certainty but may be more uncertain about the individual cluster therein. and then calculates *probabilities* associated with each cluster in this superclass based on the ratings on the HoNOS and SARN instruments. Clusters with higher probabilities are more likely to be those intended to be used in accordance with the episodic payment coding guidelines (Monitor and NHS England, 2013a, 2013b). The *best fit cluster* is defined as the cluster with the highest probability (measured in percentage points) according to the MHCT algorithm.

We examine several hospital‐level characteristics that we expect to determine hospitals' clustering behaviour.

First, for a given level of reimbursement, providers with higher cost structures face a stronger incentive to engage in discretionary coding that could inflate payment. We use detailed costing data provided by all public hospital providers in England 10All NHS providers in England are required to return detailed‐costing data annually to inform the calculation of future reimbursement tariffs (see www.gov.uk/government/collections/nhs‐reference‐costs). The reference cost data for 2013/14 are available at https://www.gov.uk/government/publications/nhs‐reference‐costs‐2013‐to‐2014. to compute the average cost per episode by hospital *h ϵ* {1, …, *H*} and superclass *jϵ*{1, 2, 3} for the year 2013/14. These data provide information on the daily costs for an admitted and nonadmitted patient day, as well as the total number of days per cluster. 11Unfortunately, data on reimbursement by cluster are not available; hence, we cannot test if the probability of upcoding increases as the difference in reimbursement between clusters increases. The average cost per episode in hospital *h* and superclass *j* in year *t* is then calculated as follows:

(1) $${\mathrm{AEC}}_{\mathrm{hjt}}=\left[\sum_{r\in j}E_{\mathrm{rht}}\left(C_{\mathrm{rht}}^{A}{\bar{D}}_{\mathrm{rht}}^{A}+C_{\mathrm{rht}}^{\mathrm{NA}}{\bar{D}}_{\mathrm{rht}}^{\mathrm{NA}}\right)\right]{\left(\sum_{r\in j}E_{\mathrm{rht}}\right)}^{-1},$$

where *E*_*rht*_ is the number of patients' episodes in cluster *r* treated in hospital *h*, $C_{\mathrm{rht}}^{A}$and $C_{\mathrm{rht}}^{\mathrm{NA}}$ are, respectively, the daily costs for admitted and nonadmitted days in cluster *r* = 1, …, *R*_*j*_ and hospital *h*, and ${\bar{D}}_{\mathrm{rht}}^{A}$ and ${\bar{D}}_{\mathrm{rht}}^{\mathrm{NA}}$ are, respectively, the average number of admitted and nonadmitted days in cluster *r* and hospital *h*. To account for the possible non‐linear effects of costs on clustering behaviour, we split the average cost per episode variable into terciles of its distribution.

Second, many providers will contract with a number of purchasers (Clinical Commissioning Groups [CCGs]), each of which can negotiate their own prices for clusters. We hypothesise that providers with more concentrated contracting relationships find it easier to tailor their coding behaviour to maximise payments, an argument similar to that made by Fernandez, McGuire, and Pradou (2017). But it may also be that providers with more, and smaller contracts may engage more in discretionary coding as they believe monitoring to be less intense. We approximate concentration of contractual arrangements by the percentage of a provider's patients that are covered by the main CCG, that is, the one representing the most of patients in the previous financial year (*t‐1* = 2013/14).

Third, coding behaviour might be related to experience, 12This is similar to the volume‐outcome relationship that has been reported for many health care procedures in which providers learn with volume so that higher past volumes generate better outcomes for current patients (Gaynor, Seider, & Vogt, 2005). which we capture using both the number of patients treated by the provider in year *t‐1* and a measure of staff engagement. In the absence of direct measures of staff engagement, we have used as a proxy, information collected through the 2013/14 NHS Staff Survey on staff training, learning, and development. 13See http://www.nhsstaffsurveys.com/Page/1040/Past‐Results/Staff‐Survey‐2013‐Detailed‐Spreadsheets/. Specifically, we computed the staff engagement proxy as the proportion of respondents in each MH hospital who *agreed/strongly agreed* with the following questions: “My training, learning and development has helped me to.... a)...do my job more effectively; b)...stay up‐to‐date with professional requirements; c)...deliver a better patient / service user experience.” We believe this provides a reasonable proxy for the dimension of staff engagement that is correlated with clinical coding and good operational practice.

Finally, patients living in more deprived neighbourhoods are expected to have higher levels of need, thus requiring more resources (Epstein, Stern, & Weissman, 1990). This may translate into a higher probability of deviation from the benchmark MHCT clusters. For this reason, we include the percentage of the provider's patient population belonging to the most deprived quintile of the 2010 IMD score distribution as an additional measure of need.

All hospital‐level variables other than average deprivation are measured in the year prior to our analysis period (i.e., respectively in *t‐1* = 2013/14, whereas *t* = 2014/15) to mitigate the risk of endogeneity bias due to reverse causality.

## 4 METHODS

To establish the extent to which the observed clustering of patients by providers deviates from the best fit cluster recommended by the MHCT, and whether this is associated with observed or unobserved hospital characteristics, we estimate two types of multilevel regression models (Rice & Jones, 1997, Snijders & Bosker, 2012).

First, we perform mixed‐effects logistic regression with patients *i* = 1, …, *n*_*h*_ treated by providers *h* = 1, …, *H* and:

(2) $$\mathrm{Pr}\left[Y_{\mathrm{ih}}=1\right]=\frac{\exp\left({X_{\mathrm{ih}}}^{\prime }\beta +P_{\mathrm{ih}}^{\prime }\delta +Z_{h}^{\prime }\theta +\mu_{h}\right)}{1+\exp\left({X_{\mathrm{ih}}}^{\prime }\beta +P_{\mathrm{ih}}^{\prime }\delta +Z_{h}^{\prime }\theta +\mu_{h}\right)},$$

where *Y*_*ih*_ equals 1 if the MHCT best fit cluster and observed cluster differ and 0 otherwise, *X*_*ih*_ is a vector of observed patient characteristics including gender, age, marital status, ethnicity, and local area deprivation, *P*_*ih*_ is the probability of the best fit cluster assigned by the MHCT, *Z*_*h*_ is observed provider characteristics such as volume of activity and production cost in 2013/14, and *μ*_*h*_ is a normally distributed random provider effect with 0 mean and variance *σ*^2^. The provider effect *μ*_*h*_ captures systematic variation across providers, conditional on observed patient and provider characteristics. 14We choose a random effects approach over a fixed effects approach to avoid incidental parameter bias in non‐linear models (Lancaster, 2000) and because we wish to explore the impact of observed hospital level characteristics on coding behaviour.


Second, and as an extension of the above, we utilise the fact that clusters are ordered according to the level of care required (and therefore resource use and likely reimbursement) to further differentiate mismatches into over and under clustering. Over clustering arises when the observed cluster number is higher than the cluster number suggested by the MHCT, and vice versa. We estimate multilevel multinomial logit models (Hedeker, 2003) of the form:

(3) $$\mathrm{Pr}\left[Y_{\mathrm{ih}}=k\right]=\frac{\exp\left({X_{\mathrm{ih}}}^{\prime }\beta_{k}+P_{\mathrm{ih}}^{\prime }\delta_{k}+Z_{h}^{\prime }\theta_{k}+\mu_{\mathrm{hk}}\right)}{\sum_{k=1}^{3}\exp\left({X_{\mathrm{ih}}}^{\prime }\beta_{k}+P_{\mathrm{ih}}^{\prime }\delta_{k}+Z_{h}^{\prime }\theta_{k}+\mu_{\mathrm{hk}}\right)},k=1,2,3,$$

$$\left(\begin{array}{c} \mu_{h1}\\ \mu_{h2} \end{array}\right)\sim \mathrm{BVN}\left(\left(\begin{array}{c} 0\\ 0 \end{array}\right),\left(\begin{array}{cc} \sigma_{1}^{2} & \sigma_{12}\\ \sigma_{12} & \sigma_{2}^{2} \end{array}\right)\right),$$

where *Y*_*ih*_ equals 1 in the case of under clustering, 2 in the case of over clustering, and 3 if MHCT and the observed clustering agree, which forms the base category.

To quantify the unobserved provider heterogeneity, we follow Larsen and Merlo (2005) and compute the median odds ratios (MORs) as follows:

(4) $$\mathrm{MOR}=\mathrm{med}\left\{\exp\left[{\left(2\sigma^{2}\right)}^{0.5}\times \Phi^{-1}\left(0.75\right)\right]\right\},$$

The MOR expresses the ratio of the probability of mismatch across two randomly chosen providers, with the higher probability forming the numerator. This can be compared with the odds ratios of other explanatory variables and thus helps to put the relative importance of unobserved heterogeneity into context.

We run separate analyses for each superclass. To check the robustness of our findings, we conducted three further analyses. First, we checked the impact of reordering the clusters on the basis of average episode cost (Section 5.3); second, we tested for a systematic provider effect on assignment (Section 5.4); lastly, we tested the impact of including patients who had previously been clustered (Section 5.5).

All models are estimated using Markov Chain Monte Carlo techniques (Browne, 2012) with maximum likelihood estimates as starting values obtained via iterative generalised least squares (Goldstein, 1986). To achieve stationarity, we run the Markov Chain Monte Carlo chain for 55,000 iterations and discard the first 5,000 iterations as *burn‐in* period (Draper, 2011; Geyer, 2011). To reduce autocorrelation and heteroscedasticity, we utilise the estimates of every 50th replication to compute point estimates and 95% credible intervals (CrIs). All computations are performed in MLwin 3.00 operated through the runMLwin 64‐bit routine (Leckie & Charlton, 2013) in Stata 13. 15In the Online Appendix, we also show results of mixed‐effect multinomial regressions in which we model the probability of a patient being assigned to a given MH cluster within a certain MH superclass.


## 5 RESULTS

### 5.1 Descriptive statistics

Our analysis sample consists of 148,472 patients (Table 2). The distribution of patients across clusters is highly concentrated, with at least 30% of patients in each superclass being categorised in a single cluster.

**Table 2:** Newly clustered patients by Superclass and mental health clusters in 2014/15

Nonpsychotic			Psychotic			Organic		
Clusters	Patients	%	Clusters	Patients	%	Clusters	Patients	%
1	4,471	5.12	10	5,728	33.81	18	21,246	48.00
2	6,532	7.49	11	2,686	15.86	19	16,653	37.62
3	20,998	24.06	12	1,983	11.71	20	4,571	10.33
4	27,093	31.05	13	1,538	9.08	21	1,796	4.06
5	11,860	13.59	14	2,799	16.52			
6	4,241	4.86	15	1,014	5.99			
7	7,193	8.24	16	734	4.33			
8	4,877	5.59	17	459	2.71			
Total	87,265	100		16,941	100		44,266	100

Table 3 presents descriptive statistics by superclass. Each hospital treated on average over 32,000 patients in the year prior to our analysis period, whereas the average percentage of patients in the CCG with the largest commissioning agreement with each provider is around 40% of their case‐load (range 14.4–97.6%). Of the 53 providers, only six had 75% or more of their total activity commissioned by a single CCG. The average cost per episode was highest for psychotic patients (around £6,060) and lower for nonpsychotic and organic patients (around £2,530). On average, the percentage of patients residing in the most deprived quintile of the IMD 2010 distribution was over 29%.

**Table 3:** Descriptive statistics by mental health superclass

MH hospital‐level variables (varying by MH hospital only, not by superclass)									
	Mean	*SD*	Median						
Number of hospital patients in 2013/14 (‘000 s)	32.54	13.42	29.62						
% patients from largest commissioning CCG in 2013/14	42.72	22.82	34.22						
% patients residing in most deprived quintile (hospital level)	29.53	18.04	25.77						
Staff Engagement (% staff agree or strongly agree)	69.48	3.74	70.15						

*MH hospital‐level variables (varying by MH hospital AND by superclass)*									
	Nonpsychotic			Psychotic			Organic		
	Mean	*SD*	Median	Mean	*SD*	Median	Mean	*SD*	Median
Average cost per episode in 2013/14 (in £1,000 s)	2.68	1.39	2.46	6.06	2.73	5.60	2.53	1.74	2.33
*Patient‐level variables*									

	Nonpsychotic			Psychotic			Organic		
	Mean	*SD*	Median	Mean	*SD*	Median	Mean	*SD*	Median
Female	0.58	0.49		0.47	0.50		0.61	0.49	
Age (mean)	42.19	18.87	39	42.73	19.49	39	80.24	9.58	82
age 0–18 years	0.05	0.21		0.04	0.21		—	—	
age 19–29 years	0.27	0.45		0.28	0.45		0.00	0.03	
age 30–39 years	0.18	0.39		0.18	0.39		0.00	0.04	
age 40–49 years	0.18	0.38		0.16	0.37		0.01	0.08	
age 50–59 years	0.13	0.34		0.13	0.33		0.03	0.16	
age 60–69 years	0.07	0.26		0.08	0.27		0.09	0.28	
age 70–79 years	0.06	0.23		0.06	0.24		0.28	0.45	
age over 80 years	0.05	0.22		0.06	0.23		0.60	0.49	
Ethnicity: White	0.70	0.46		0.64	0.48		0.80	0.40	
Ethnicity: Mixed	0.01	0.11		0.02	0.13		0.00	0.06	
Ethnicity: Asian	0.04	0.19		0.09	0.29		0.03	0.16	
Ethnicity: Black	0.02	0.14		0.09	0.28		0.01	0.12	
Ethnicity: Other	0.23	0.42		0.17	0.37		0.16	0.37	
Marital Status: Divorced	0.04	0.19		0.04	0.20		0.04	0.19	
Marital Status: Married	0.19	0.39		0.18	0.38		0.35	0.48	
Marital Status: Undisclosed	0.33	0.47		0.24	0.43		0.30	0.46	
Marital Status: Separated	0.03	0.16		0.02	0.15		0.01	0.08	
Marital Status: Single	0.37	0.48		0.47	0.50		0.06	0.24	
Marital Status: Widowed	0.04	0.19		0.04	0.20		0.26	0.44	
IMD 2010 1st quintile (least deprived)	0.13	0.34		0.10	0.30		0.20	0.40	
IMD 2010 2nd quintile	0.16	0.37		0.13	0.34		0.21	0.41	
IMD 2010 3rd quintile	0.20	0.40		0.17	0.38		0.21	0.41	
IMD 2010 4th quintile	0.23	0.42		0.24	0.43		0.19	0.39	
IMD 2010 5th quintile (most deprived)	0.28	0.45		0.35	0.48		0.18	0.38	
Total HoNOS score (0–48)	12.74	5.93	12	14.98	6.80	15	11.07	5.89	10
Total SARN score (0–24)	3.65	3.64	3	5.27	4.00	5	2.11	2.85	1
Proportion with total SARN score >0	0.74	0.44	1	0.86	0.35	1	0.54	0.50	1
Probability of MHCT *best fit cluster*	28.53	8.18	26.43	17.37	2.36	16.73	43.55	8.27	42.98

*Note*. CCG: Clinical Commissioning Group; HoNOS: Health of the Nation Outcomes Scales; IMD: Index of Multiple Deprivation; MH: mental health; MHCT: mental health clustering tool; SARN: Summary Assessment of Risk and Need. The analysis includes patients treated in 53 mental health hospitals in England in the nonpsychotic Superclass, and 52 in the psychotic and organic superclasses.

We estimate the polychoric correlations between current and past cluster assignments for each episode with multiple clusters, using the full sample of MH patients treated in year 2014/15. 16These correlations are estimated in Stata by using the *polychoric* user‐written function (Kolenikov & Angeles, 2004), that provides a more accurate estimate than the Stata built‐in function (Uebersax, 2015). The correlations (standard errors in parenthesis) are 0.5826 (0.001615), 0.5936 (0.001599), and 0.5956 (0.001596) for clusters in the nonpsychotic, psychotic, and organic superclasses, respectively, which establishes that cluster assignment is persistent within each superclass.

Table 4 compares the Best Fit cluster allocation (rows) suggested by the MHCT algorithm with the observed cluster allocation (columns). The diagonal indicates the proportion of patients where suggested and observed allocations coincide. The average agreement across the 20 clusters is 35.9%, with only four cells showing agreement in excess of 50%. The weighted kappa statistic, a measure of agreement that penalises according to the degree of mismatch (Cohen, 1968), is equal to 0.3759 (0.0020) in the nonpsychotic superclass subsample, and respectively 0.2022 (0.0042) and 0.4675 (0.0034) for the psychotic and organic superclass subsamples, suggesting slight to moderate agreement (Landis & Koch, 1977). 17The weights are defined in the standard way as 1 − |*r* − *d*|/(*m* − 1), where *r* and *d* index the rows and columns of the clusters by the different assignment mechanisms (MHCT algorithm or clinician) within each superclass, and *m* is the maximum number of possible clusters within each superclass.


**Table 4:** Differences in assignment to mental health Cluster: mental health clustering tool algorithm versus Clinician assignment (row‐wise percentages)

		Cluster assigned by clinician at first clustering								
Recommended cluster (from running MHCT algorithm)		**Superclass: Nonpsychotic**								
		1	2	3	4	5	6	7	8	**Number of patients**
	**1**	22.51%	17.73%	28.92%	16.98%	5.40%	1.66%	3.82%	2.99%	6,149
	**2**	19.82%	34.69%	18.70%	15.34%	5.40%	1.78%	2.11%	2.17%	1,519
	**3**	7.80%	12.34%	46.98%	19.98%	5.33%	1.25%	4.10%	2.22%	22,282
	**4**	2.55%	5.26%	19.10%	50.09%	12.08%	2.02%	6.00%	2.90%	28,499
	**5**	0.96%	1.33%	5.12%	25.25%	50.50%	4.48%	7.56%	4.80%	7,992
	**6**	0.68%	1.61%	6.48%	19.16%	23.53%	26.01%	14.82%	7.71%	4,399
	**7**	1.51%	3.39%	15.63%	26.99%	7.92%	15.21%	24.09%	5.27%	9,033
	**8**	1.06%	2.49%	12.46%	24.19%	13.95%	5.17%	11.76%	28.94%	7,392
		**Superclass: Psychotic**								
		**10**	**11**	**12**	**13**	**14**	**15**	**16**	**17**	**Number of patients**
	**10**	48.32%	6.86%	7.81%	5.79%	16.55%	4.58%	9.29%	0.81%	743
	**11**	28.00%	40.28%	10.97%	3.89%	8.63%	2.20%	3.42%	2.61%	2,954
	**12**	36.72%	19.22%	23.11%	4.67%	9.52%	3.21%	1.49%	2.04%	3,423
	**13**	41.49%	8.00%	7.69%	16.69%	21.68%	3.01%	0.50%	0.95%	4,225
	**14**	24.30%	4.99%	8.39%	14.61%	37.31%	4.48%	0.87%	5.06%	1,383
	**15**	30.30%	8.42%	7.90%	7.12%	12.70%	28.81%	2.17%	2.58%	1,937
	**16**	29.12%	7.52%	6.63%	5.67%	20.16%	1.30%	27.27%	2.32%	1,463
	**17**	22.51%	13.16%	14.64%	11.32%	15.01%	4.80%	4.80%	13.78%	813
		**Superclass: Organic**								
		**18**		**19**		**20**		**21**		**Number of patients**
	**18**	74.89%		23.09%		1.68%		0.34%		20,337
	**19**	29.31%		57.54%		10.68%		2.47%		16,459
	**20**	16.76%		36.76%		35.43%		11.05%		4,905
	**21**	14.42%		26.67%		28.58%		30.33%		2,565

*Note*. MHCT: mental health clustering tool. Rows: recommended clusters according to the MHCT algorithm. Columns: clusters assigned by clinician at first clustering. The superclass used in this tabulation is the one implied by the mental health cluster recorded in the Mental Health Services Data Set. Each cell reports the percentage of patients classified in a given cluster by the clinician(s) as a proportion of the total number of patients assigned to the same cluster by the MHCT algorithm (total by row).

The match between observed and suggested cluster allocation varies across the superclasses. The highest average match is observed for the organic superclass with 49.6% (range 30.3–74.9%), possibly because there are fewer groups, whereas the lowest average match is observed for the psychotic superclass (29.5%; range 13.8–48.3%). In those instances where observed and suggested cluster allocation are in disagreement, the observed cluster is usually adjacent to that suggested, though with no clear direction, and the probability of observed assignment decreases with the distance between observed and suggested cluster. A noteworthy exception is the psychotic superclass in which patients are likely to be assigned to cluster 10 (“First episode in psychosis”) independent of the severity of the suggested cluster, that is, the distance between classes. 18Cluster 10 is a specific clinical presentation, which is usually treated in early intervention in psychosis teams and not all patients who develop psychosis will necessarily start in cluster 10.


### 5.2 Determinants of mismatch between observed and suggested cluster allocation

Table 5 shows the main regression results. Columns 1–3 report results for Equation (2), which models the probability of mismatch between the clinician and the MHCT algorithm. 19In Online Appendix Table B.I., we show the results of the same binary logit mixed effect regression model, when only hospital random effects are included, and then additional covariates are sequentially added. Columns 4–9 report results for Equation (3), which further distinguishes between over clustering (patients are assigned to a higher cluster than suggested by the MHCT) and under clustering. The reference category in each analysis is agreement between clinician and MHCT algorithm.

**Table 5:** Mismatch between clinician assignment and mental health clustering tool algorithm assignment, mixed‐effects multilevel multinomial logit model

	Mixed‐effects binary logit model			Mixed‐effects multinomial logit model					
Odds ratio	Nonpsychotic	Psychotic	Organic	Nonpsychotic		Psychotic		Organic	
	1	2	3	4	5	6	7	8	9
	Odds ratio: unmatched vs. matched	Odds ratio: unmatched vs. matched	Odds ratio: unmatched vs. matched	Odd ratio: underclustered vs. matched	Odd ratio: overclustered vs. matched	Odd ratio: underclustered vs. matched	Odd ratio: overclustered vs. matched	Odd ratio: underclustered vs. matched	Odd ratio: overclustered vs. matched
Total HoNOS score	0.9827*** (−11.799)	1.0105*** (3.449)	1.0850*** (38.594)	0.9837*** (−9.616)	0.9819*** (−10.110)	1.0138*** (4.301)	0.9994 (−0.151)	1.1080*** (39.832)	1.0493*** (17.247)
Total SARN score^a^	1.0781*** (29.968)	1.0046 (0.887)	1.2071*** (7.778)	1.1189*** (39.623)	1.0267*** (8.405)	1.0084 (1.523)	0.9862** (−2.013)	1.3123*** (8.966)	1.0695** (2.168)
Probability of MHCT *best fit cluster*	0.9725*** (−30.474)	0.9411*** (−7.882)	0.9817*** (−14.834)	0.9692*** (−29.113)	0.9756*** (−21.168)	0.9837** (−2.094)	0.7255*** (−19.290)	0.9946*** (−3.961)	0.9419*** (−24.295)
Number of hospital patients in 2013/14	0.9912 (−1.401)	0.9932 (−1.170)	0.9849*** (−3.024)	0.9882 (−1.508)	0.9918 (−1.452)	0.9943 (−0.975)	0.9937 (−1.075)	0.9818*** (−2.715)	0.9897* (−1.930)
% patients from largest CCG in 2013/14	1.0019 (0.502)	1.0009 (0.274)	0.9991 (−0.285)	1.0032 (0.714)	0.9983 (−0.467)	1.0026 (0.693)	0.9974 (−0.698)	0.9964 (−0.864)	1.0010 (0.310)
1st tercile of average cost per episode in 2013/14	0.8293 (−1.132)	0.7261** (−2.134)	0.7980 (−1.563)	0.7495 (−1.341)	0.9946 (−0.029)	0.7160** (−2.113)	0.7718 (−1.591)	0.8155 (−1.055)	0.7932 (−1.458)
3rd tercile of average cost per episode in 2013/14	0.8358 (−0.976)	0.8595 (−0.979)	0.9514 (−0.341)	0.7247 (−1.560)	0.9387 (−0.415)	0.8875 (−0.737)	0.8356 (−1.078)	0.7821 (−1.305)	1.0561 (0.353)
% most deprived quintile patients (hospital level)	1.0255*** (2.597)	1.0119 (1.563)	1.0074 (1.087)	1.0214** (1.984)	1.0194** (2.378)	1.0154* (1.868)	1.0059 (0.706)	1.0012 (0.116)	1.0135* (1.673)
Staff engagement (% agree or more)	0.9880 (−0.557)	1.0011 (0.066)	0.9793 (−1.334)	0.9653 (−1.163)	1.0200 (0.886)	0.9964 (−0.192)	1.0107 (0.571)	0.9690 (−1.349)	0.9895 (−0.579)
ICC (intraclass correlation coefficient)	0.0761	0.0474	0.0433	0.1160	0.0717	0.0548	0.0521	0.0750	0.0473
MOR (median odds ratio)	1.6432	1.4712	1.4448	1.8717	1.6172	1.5170	1.5003	1.6365	1.4706
MOR 95% Cred. Int. L. Bound	1.5055	1.3612	1.3393	1.6640	1.4704	1.3928	1.3702	1.4820	1.3565
MOR 95% Cred. Int. U. Bound	1.8266	1.6404	1.5907	2.1643	1.7994	1.6802	1.6724	1.8615	1.6237
Probability (unmatched assignment)	0.6306	0.8319	0.3621	0.3405	0.2815	0.6510	0.1739	0.1734	0.1823
Prob. (unmatched assignment) 95% Cred. Int. L. Bound	0.5703	0.7957	0.3139	0.2868	0.2393	0.5984	0.1409	0.1362	0.1495
Prob. (unmatched assignment) 95% Cred. Int. U. Bound	0.6870	0.8677	0.4128	0.4036	0.3256	0.6978	0.2152	0.2149	0.2203
Observations	87,265	16,941	44,266	87,265		16,941		44,266	
Time	7,533	1,428	3,383	22,927		5,072		9,991	
DIC	110,577	18,920	55,273	174,907		32,100		76,280	

*Note*. CCG: Clinical Commissioning Group; HoNOS: Health of the Nation Outcomes Scales; MHCT: mental health clustering tool; SARN: Summary Assessment of Risk and Need. Columns 1–3: Unmatched = Cluster assigned by clinician is different than cluster assigned by MHCT algorithm. Columns 4–9: Unmatched = cluster assigned by clinician is higher (overclustered) or lower (underclustered) than Cluster assigned by MHCT algorithm. Nonpsychotic and psychotic patients reference categories are the following: Male, aged 19–29, White, single, most deprived. Organic patients reference categories are the following: Male, aged over 80, white, married, most deprived. Burn‐in: 5,000; Chain: 50,000; Thinning: 50; *t* statistics in parentheses.

* *p* < 0.10.

** *p* < 0.05.

*** *p* < 0.01.

a Organic superclass: Variable is a binary dummy = 1 if total SARN score >0.

Focussing on the first analysis (first three columns of Table 5), in each superclass, the probability of a mismatch is negatively correlated with the probability of the Best Fit cluster suggested by the MHCT (significant at *p* < 0.01). This suggests that clinicians and the algorithm respond to similar signals of severity so that the degree of discretionary coding reduces as the uncertainty about cluster allocation reduces. However, the relationship is not perfect: the average marginal effect 20The average marginal effect is calculated as the change in the probability of mismatch for a 1‐percentage point increase in the independent variable of interest holding all other covariates at their observed level; averaged across all observations in the sample (Cameron & Trivedi, 2010). of a percentage point increase in the probability of Best Fit cluster (that is measured in percentage points) is associated with a 0.64% decrease (*SE*: 0.0002) in the probability of mismatch in the nonpsychotic subsample, and with a decrease in the probability of mismatch of 1.13% (*SE*: 0.0014) for psychotic patients and a decrease of 0.43% (*SE*: 0.0003) for organic patients, respectively.

In the nonpsychotic superclass, higher cumulative HoNOS scores are associated with lower probability of a mismatch, and this holds true for over and under clustering alike. Conversely, for patients in the psychotic and organic superclasses, the models estimate a positive association between cumulative HoNOS score and mismatch. For the first patient group, the association is driven by an increased probability of under clustering but not over clustering. Higher SARN scores are associated with a higher probability of mismatch for patients in the nonpsychotic and organic superclasses.

Only a few provider characteristics are statistically significantly associated with the probability of mismatch. Providers with higher volumes of activity in the past year are less likely to diverge from the MHCT suggestion, although the effect is only statistically significant for the organic superclass. The proportion of a provider's patients residing in the most deprived areas of the country is associated with a lower probability of mismatch for the nonpsychotic superclass, and a lower probability of over clustering (but not under clustering) for the organic superclass. Average cost and contractual homogeneity are not associated with divergent coding behaviour for any superclass at the 5% level.

The estimated MORs reveal the existence of substantive unexplained between‐hospital variability in coding behaviour. Using results from Equation (2); columns 1–3 in Table 5), in two randomly selected hospitals, the probability of a given patient being clustered differently by the clinician and the MHCT algorithm is approximately 45–64% higher in one hospital than the other. These effects are large in comparison to the effects of observable patient and provider characteristics. For example, in the organic superclass (column 3) increasing a hospital's activity by 10,000 patients or increasing a patient's HoNOS score by 10 points leads to an increased risk of mismatch of 14% and 8.5%, respectively. Using results from Equation (3); columns 4–9 in Table 5), we find that provider heterogeneity is more pronounced in the probability of under clustering than over clustering but that this difference is not statistically significantly different from zero as indicated by overlapping 95% CrIs.

### 5.3 Robustness check: Mismatch when clusters are ordered by average cost per episode

One potential drawback in our empirical analysis is the assumption that clusters are ordered according to the level of care required, and therefore also by their expected reimbursement. 21The binary mixed effect logit regression model shown in Table 5, columns 1–3, and the multinomial mixed effect logit regression models shown in Online Appendix Tables C.I–III are not affected by the assumed cluster order. However, the order based on reference unit costs of the clusters is not the same as the clusters' nominal order. To test whether our results on the quantification of the coding discretion are robust to the cluster ordering, we reorder the clusters within each superclass based on the average cost per episode across all hospitals in year 2013/14, (see Equation (1), Section 3), and we reestimate the models in Table 5 using the new cluster order based on such average costs. 22By using the average cost per episode across all hospitals in year 2013/14 as shown by Equation 1, the ordering of clusters based on reference costs takes into account the fact that the effective cost per cluster is a weighted average of the costs of both admitted and nonadmitted patients that are treated by the hospital. The last two columns of Table 1 report the average cost per episode of each cluster and the clusters' order based on such average costs. The original ordering is almost unchanged for nonpsychotic clusters; it is exactly the same in the organic superclass, and shows several changes in the clusters of the psychotic superclass. The results of the new estimation are presented in Table 6 and show that although the significance level of some of the regression odd ratios coefficients (HoNOS and SARN scores) for mismatching in the psychotic superclass change (but not their magnitude; compare columns 6 and 7 of Tables 5 and 6), both the point estimates of the MORs and their 95% CrIs remain largely unchanged. The other two superclasses are not affected by the change. Overall, these findings are reassuring about the robustness of our results on discretionary hospital coding with respect to the ordering of the clusters.

**Table 6:** Mismatch between clinician assignment and mental health clustering tool algorithm assignment, with clusters ordered by average costs per episode, mixed‐effects multilevel multinomial logit model

	Mixed‐effects binary logit model			Mixed‐effects multinomial logit model					
Odds ratio	Nonpsychotic	Psychotic	Organic	Nonpsychotic		Psychotic		Organic	
	1	2	3	4	5	6	7	8	9
	Odds ratio: unmatched vs. matched	Odds ratio: unmatched vs. matched	Odds ratio: unmatched vs. matched	Odd ratio: underclustered vs. matched	Odd ratio: overclustered vs. matched	Odd ratio: underclustered vs. matched	Odd ratio: overclustered vs. matched	Odd ratio: underclustered vs. matched	Odd ratio: overclustered vs. matched
Total HoNOS score	0.9827*** (−11.799)	1.0105*** (3.449)	1.0850*** (38.594)	0.9828*** (−10.209)	0.9828*** (−9.708)	1.0406*** (11.583)	0.9769*** (−6.682)	1.1080*** (39.832)	1.0493*** (17.247)
Total SARN score‡	1.0781*** (29.968)	1.0046 (0.887)	1.2071*** (7.778)	1.1138*** (37.599)	1.0344*** (11.037)	1.0153*** (2.704)	0.9915 (−1.448)	1.3123*** (8.966)	1.0695** (2.168)
Probability of MHCT *best fit cluster*	0.9725*** (−30.474)	0.9411*** (−7.882)	0.9817*** (−14.834)	0.9741*** (−25.090)	0.9693*** (−26.286)	0.9495*** (−6.236)	0.9147*** (−9.058)	0.9946*** (−3.961)	0.9419*** (−24.295)

Number of hospital patients in 2013/14	0.9912 (−1.401)	0.9932 (−1.170)	0.9849*** (−3.024)	0.9914 (−1.180)	0.9933 (−1.064)	0.9948 (−0.808)	0.9937 (−1.094)	0.9818*** (−2.715)	0.9897* (−1.930)
% patients from largest CCG in 2013/14	1.0019 (0.502)	1.0009 (0.274)	0.9991 (−0.285)	1.0035 (0.801)	0.9987 (−0.371)	1.0028 (0.702)	1.0001 (0.017)	0.9964 (−0.864)	1.0010 (0.310)

1st tercile of average cost per episode in 2013/14	0.8293 (−1.132)	0.7261** (−2.134)	0.7980 (−1.563)	0.7919 (−0.970)	0.9684 (−0.174)	0.7158** (−2.002)	0.7694* (−1.761)	0.8155 (−1.055)	0.7932 (−1.458)
3rd tercile of average cost per episode in 2013/14	0.8358 (−0.976)	0.8595 (−0.979)	0.9514 (−0.341)	0.8093 (−0.829)	0.9843 (−0.082)	0.8156 (−1.171)	0.8885 (−0.755)	0.7821 (−1.305)	1.0561 (0.353)
% most deprived quintile patients (hospital level)	1.0255*** (2.597)	1.0119 (1.563)	1.0074 (1.087)	1.0237* (1.923)	1.0218** (2.390)	1.0148* (1.791)	1.0090 (1.218)	1.0012 (0.116)	1.0135* (1.673)
Staff engagement (% agree or more)	0.9880 (−0.557)	1.0011 (0.066)	0.9793 (−1.334)	0.9599 (−1.539)	1.0184 (0.946)	0.9905 (−0.485)	1.0175 (0.993)	0.9690 (−1.349)	0.9895 (−0.579)
ICC (intraclass correlation coefficient)	0.0761	0.0474	0.0433	0.1245	0.0692	0.0611	0.0460	0.0750	0.0473
MOR (median odds ratio)	1.6432	1.4712	1.4448	1.9203	1.6028	1.5546	1.4619	1.6365	1.4706
MOR 95% Cred. Int. L. Bound	1.5055	1.3612	1.3393	1.4183	1.3505	1.4183	1.3505	1.4820	1.3565
MOR 95% Cred. Int. U. Bound	1.8266	1.6404	1.5907	1.7400	1.6213	1.7400	1.6213	1.8615	1.6237
Probability (unmatched assignment)	0.6306	0.8319	0.3621	0.3460	0.3616	0.3764	0.4490	0.1734	0.1823
Prob. (unmatched assignment) 95% Cred. Int. L. Bound	0.5703	0.7957	0.3139	0.2906	0.3058	0.3247	0.3984	0.1362	0.1495
Prob. (unmatched assignment) 95% Cred. Int. U. Bound	0.6870	0.8677	0.4128	0.4107	0.4200	0.4282	0.4987	0.2149	0.2203
Observations	87,265	16,941	44,266	87,265		16,941		44,266	
Time	7,533	1,428	3,383	22,359		4,298		9,991	
DIC	110,577	18,920	55,273	175,155		35,014		76,280	

*Note*. CCG: Clinical Commissioning Group; HoNOS: Health of the Nation Outcomes Scales; MHCT: mental health clustering tool; SARN: Summary Assessment of Risk and Need. Columns 1–3: Unmatched = Cluster assigned by clinician is different than cluster assigned by MHCT algorithm. Columns 4–9: Unmatched = cluster assigned by Clinician is higher (overclustered) or lower (underclustered) than cluster assigned by MHCT algorithm. Nonpsychotic and psychotic patients reference categories are: Male, aged 19–29, White, single, most deprived. Organic patients reference categories are: Male, aged over 80, white, married, most deprived. Burn‐in: 5,000; Chain: 50,000; Thinning: 50; *t* statistics in parentheses.

* *p* < 0.10.

** *p* < 0.05.

*** *p* < 0.01.

‡ Organic superclass: Variable is a binary dummy = 1 if Total SARN score >0.

### 5.4 Robustness check: Provider effect on patients' assignment to individual clusters

We also investigate the presence of a systematic provider effect on patients' assignment to individual clusters to test whether providers disagree in their assignment to specific clusters (see Online Appendix C). Provider heterogeneity is somewhat more pronounced in this analysis than when assessing mismatch, as evidenced by the larger variation in MORs from 1.46 to 3.59 across clusters in different superclasses. However, MORs are broadly similar across clusters in the same superclass, suggesting that the allocation of patients to some clusters rather than others suggesting these is less heterogeneity across providers, once the patient's prevalent MH disorder, identified by the assigned superclass, has been determined.

### 5.5 Robustness check: Determinants of mismatch using the full sample

In Online Appendix Tables D.I and D.II, we present the estimation results of the regression models investigating the mismatch in the patient assignment, without imposing the restriction of patients not having been previously clustered. We use a 50% clustered random sample (with clustering by MH hospitals and MH clusters) of the original sample of patients treated in year 2014/15. 23The full sample is too large to be analysed in MLwin. Nevertheless, the 50% clustered random sample should provide unbiased, albeit slightly less precise results. The results for the MORs are either very similar to the ones provided in Tables 5 and 6 (with clusters ordered by average costs), or the 95% CrI of the two sets of estimates (with and without the “newly clustered patient” restriction) overlap at least partially, suggesting once again the robustness of our findings.

## 6 DISCUSSION AND CONCLUSIONS

The English NHS is moving to a new reimbursement model for mental health care that links payment to activity, thus aligning the payment system in MH to those common in many physical health care systems. Although this change may help create a fair and sustainable funding system, there are also well‐known risks of unintended consequences such as incentives to inappropriately allocate patients higher payment groups.

We have examined the extent to which the categorisation of NHS patients by MH providers is subject to discretion. For this purpose, we investigated differences between patients' first cluster assignments by clinicians and those assignments suggested by an external standard, the MHCT algorithm. We find MORs ranging from 1.46 to 1.88, which reflects significant unexplained variation between providers in how they allocate patients to clusters over and above observed need factors. Unobserved provider effects are at least as important as observed hospital characteristics such as volume or deprivation in determining cluster allocation. Variations between providers may be driven by differences in severity, treatment thresholds, or subjective perceptions in recording of HoNOS scores. Some of the predictors in our model may be suggestive of discretionary behaviour, for example, where they reflect resource pressures, although others may be more indicative of broader aspects impacting on service delivery, for example, levels of deprivation, though these may also indirectly affect decisions around levels of care intensity. However, the observed discretionary behaviour may not be associated with attracting higher payments due to financial considerations because average costs were not associated with upcoding. Furthermore, we do not find evidence that provider differences are more likely to result in upcoding than downcoding.

Our study has a number of limitations. First, it is possible that there may be legitimate unobservable differences between providers that determine their allocation behaviour that we have not been able to account for. In this case, the observed MORs capture discretionary behaviour as well as case‐mix differences and external constraints, although it is *a priori* unclear whether this leads to inflated or deflated estimates of the MOR. Second, although the MHCT has been recommended as a guide for clustering, its use may differ across providers and this would also be captured by the MOR. Unfortunately, use of the MHCT is not recorded, so we cannot explore this further. Finally, throughout the study period providers were required to cluster patients but reimbursement was not linked to cluster allocation at this time. Hence, our analysis should be understood as exploring the *potential* for discretionary coding, rather than as evidence that providers respond to incentives by exploiting the flexibility granted by the classification system.

The considerable degree of discretion in the English MH clustering system has important implications for policymakers in the design and operation of the payment system. Clinical judgement may play a larger role in allocation within the MH context than in acute care where diagnostic information and procedures may be more clear‐cut and, hence, auditable. Nevertheless, those responsible for the design of the MH payment system will need to find ways to put checks in place to ensure the integrity and fairness of the reimbursement model. The MHCT may offer a starting point, and providers could be required to justify deviations from the proposed cluster allocation if the level of mismatch breaches certain thresholds. This would require continued development and validation of the MHCT algorithm to ensure that it generates consistent groupings of patients with similar needs (Jacobs, 2014).

## Supporting information

Data S1. Table AI. HoNOS and SARN items, and their scoring
Table B.I. Probability of Mismatch between clinician assignment and MHCT algorithm assignment ‐ Mixed‐effects Binary Logit Model: successive entry of predictors (to be compared with Table V in the main manuscript).
Table C.I. Mixed‐effects multilevel multinomial logit model – Investigating provider effects in assigning patients to specific clusters in the non‐psychotic superclass.
Table C.II. Mixed‐effects multilevel multinomial logit model – Investigating provider effects in assigning patients to specific clusters in the psychotic superclass.
Table C.III. Mixed‐effects multilevel multinomial logit model – Investigating provider effects in assigning patients to specific clusters in the organic superclass.
Table D.I. Mismatch between clinician assignment and MHCT algorithm assignment. Mixed‐effects multilevel multinomial logit model ‐ 50% random draws from the full sample (to be compared with Table V in the main manuscript).
Table D.II. Mismatch between clinician assignment and MHCT algorithm assignment, with clusters order on average costs per episode. Mixed‐effects multilevel multinomial logit model – 50% random draws from the full sample (to be compared with Table VI in the main manuscript).
