# Supplementary material for: Prospective payment systems and discretionary coding—Evidence from English mental health providers
Source: Health Econ. 2018 Dec 27;28(3):387–402. doi: 10.1002/hec.3851 (PMC6491985; doi:10.1002/hec.3851)
Supplement: Supplementary file 1 — Data S1. Table AI. HoNOS and SARN items, and their scoring Table B.I. Probability of Mismatch between clinician assignment and MHCT algorithm assignment ‐ Mixed‐effects Binary Logit Model: successive entry of predictors (to be compared with Table V in the main manuscript). Table C.I. Mixed‐effects multilevel multinomial logit model – Investigating provider effects in assigning patients to specific clusters in the non‐psychotic superclass. Table C.II. Mixed‐effects multilevel multinomial logit model – Investigating provider effects in assigning patients to specific clusters in the psychotic superclass. Table C.III. Mixed‐effects multilevel multinomial logit model – Investigating provider effects in assigning patients to specific clusters in the organic superclass. Table D.I. Mismatch between clinician assignment and MHCT algorithm assignment. Mixed‐effects multilevel multinomial logit model ‐ 50% random draws from the full sample (to be compared with Table V in the main manuscript). Table D.II. Mismatch between clinician assignment and MHCT algorithm assignment, with clusters order on average costs per episode. Mixed‐effects multilevel multinomial logit model – 50% random draws from the full sample (to be compared with Table VI in the main manuscript). [file HEC-28-387-s001.docx]

# Appendix

(Appendices to the paper “*Prospective payment systems and discretionary coding - Evidence from English mental health providers*” by Giuseppe Moscelli, Rowena Jacobs, Nils Gutacker, María José Aragón, Martin Chalkley, Anne Mason, Jan R. Böhnke).

## A. HoNOS, SARN and their scoring

**Table A.I.** **HoNOS and SARN items, and their scoring**

| **HoNOS Item** |
| --- |
| 1. Overactive, aggressive, disruptive or agitated behaviour |
| 1. Non-accidental self-injury |
| 1. Problem-drinking or drug-taking |
| 1. Cognitive problems |
| 1. Physical illness or disability problems |
| 1. Problems associated with hallucinations and delusions |
| 1. Problems with depressed mood |
| 1. Other mental and behavioural problems |
| 1. Problems with relationships |
| 1. Problems with activities of daily living |
| 1. Problems with living conditions |
| 1. Problems with occupation and activities 2. Strong unreasonable beliefs that are not psychotic in origin |
| **SARN Item** |
| 1. Agitated behaviour/expansive mood (historical) |
| 1. Repeat self-harm (historical) |
| 1. Safeguarding other children & vulnerable adults (historical) |
| 1. Engagement (historical) |
| 1. Vulnerability (historical) |
| **Scoring** |
| 1. No problem |
| 1. Minor problem requiring no action |
| 1. Mild problem but definitely present |
| 1. Moderately severe problem |
| 1. Severe to very severe problem |

## Sequential inclusion of covariates (mixed effect logit model).

**Table B.I. Probability of Mismatch between clinician assignment and MHCT algorithm assignment - Mixed-effects Binary Logit Model: successive entry of predictors (to be compared with Table V in the main manuscript).**

|  | **Non-psychotic** | **Non-psychotic** | **Non-psychotic** | **Non-psychotic** | **Psychotic** | **Psychotic** | **Psychotic** | **Psychotic** | **Organic** | **Organic** | **Organic** | **Organic** |
| --- | --- | --- | --- | --- | --- | --- | --- | --- | --- | --- | --- | --- |
| **Odds Ratio (unmatched VS matched)** | **1** | **2** | **3** | **4** | **5** | **6** | **7** | **8** | **9** | **10** | **11** | **12** |
| Constant | 1.6011*** | 1.6488*** | 3.3587*** | 7.9780 | 3.0146*** | 4.4592*** | 10.7782*** | 12.0958** | 0.7089*** | 0.7557*** | 0.9140 | 4.1788 |
|  | (6.210) | (6.171) | (14.391) | (1.355) | (17.863) | (16.342) | (15.044) | (2.030) | (-5.204) | (-3.889) | (-0.322) | (1.237) |
| Total HONOS score |  |  | 0.9828*** | 0.9827*** |  |  | 1.0105*** | 1.0105*** |  |  | 1.0850*** | 1.0850*** |
|  |  |  | (-11.637) | (-11.799) |  |  | (3.443) | (3.449) |  |  | (38.661) | (38.594) |
| Total SARN score ‡ |  |  | 1.0782*** | 1.0781*** |  |  | 1.0049 | 1.0046 |  |  | 1.2064*** | 1.2071*** |
|  |  |  | (30.018) | (29.968) |  |  | (0.954) | (0.887) |  |  | (7.864) | (7.778) |
| Probability of MHCT Best Fit Cluster |  |  | 0.9725*** | 0.9725*** |  |  | 0.9411*** | 0.9411*** |  |  | 0.9817*** | 0.9817*** |
|  |  |  | (-30.192) | (-30.474) |  |  | (-8.010) | (-7.882) |  |  | (-14.724) | (-14.834) |
| Number of hospital patients in 2013/14 |  |  |  | 0.9912 |  |  |  | 0.9932 |  |  |  | 0.9849*** |
|  |  |  |  | (-1.401) |  |  |  | (-1.170) |  |  |  | (-3.024) |
| % patients from largest CCG in 2013/14 |  |  |  | 1.0019 |  |  |  | 1.0009 |  |  |  | 0.9991 |
|  |  |  |  | (0.502) |  |  |  | (0.274) |  |  |  | (-0.285) |
| 1^st^ tercile of average cost per episode in 2013/14 |  |  |  | 0.8293 |  |  |  | 0.7261** |  |  |  | 0.7980 |
|  |  |  |  | (-1.132) |  |  |  | (-2.134) |  |  |  | (-1.563) |
| 3^rd^ tercile of average cost per episode in 2013/14 |  |  |  | 0.8358 |  |  |  | 0.8595 |  |  |  | 0.9514 |
|  |  |  |  | (-0.976) |  |  |  | (-0.979) |  |  |  | (-0.341) |
| % most deprived quintile patients (hospital-level) |  |  |  | 1.0255*** |  |  |  | 1.0119 |  |  |  | 1.0074 |
|  |  |  |  | (2.597) |  |  |  | (1.563) |  |  |  | (1.087) |
| Staff Engagement (% agree or more) |  |  |  | 0.9880 |  |  |  | 1.0011 |  |  |  | 0.9793 |
|  |  |  |  | (-0.557) |  |  |  | (0.066) |  |  |  | (-1.334) |
| ICC (Intraclass Correlation Coefficient) | 0.0826 | 0.0841 | 0.0821 | 0.0761 | 0.0494 | 0.0514 | 0.0517 | 0.0474 | 0.0608 | 0.0605 | 0.0433 | 0.0433 |
| MOR (Median Odds Ratio) | 1.6808 | 1.6892 | 1.6780 | 1.6432 | 1.4837 | 1.4956 | 1.4978 | 1.4712 | 1.5532 | 1.5513 | 1.4449 | 1.4448 |
| MOR 95% Cred. Int. L. Bound | 1.5266 | 1.5361 | 1.5244 | 1.5055 | 1.3720 | 1.3767 | 1.3886 | 1.3612 | 1.4260 | 1.4285 | 1.3441 | 1.3393 |
| MOR 95% Cred. Int. U. Bound | 1.8890 | 1.8737 | 1.8797 | 1.8266 | 1.6308 | 1.6410 | 1.6535 | 1.6404 | 1.7309 | 1.7185 | 1.5919 | 1.5907 |
| Probability (unmatched assignment) | 0.6140 | 0.6219 | 0.6203 | 0.6306 | 0.7508 | 0.8160 | 0.8238 | 0.8319 | 0.4153 | 0.4306 | 0.4831 | 0.3621 |
| Prob. (unmatched assignment) 95% Cred. Int. L. Bound | 0.5805 | 0.5843 | 0.5841 | 0.5703 | 0.7250 | 0.7852 | 0.7963 | 0.7957 | 0.3852 | 0.3950 | 0.3548 | 0.3139 |
| Prob. (unmatched assignment) 95% Cred. Int. U. Bound | 0.6465 | 0.6591 | 0.6565 | 0.6870 | 0.7742 | 0.8417 | 0.8490 | 0.8677 | 0.4477 | 0.4659 | 0.6085 | 0.4128 |
| Observations | 87265 | 87265 | 87265 | 87265 | 16941 | 16941 | 16941 | 16941 | 44266 | 44266 | 44266 | 44266 |
| Time | 756 | 5642 | 6239 | 7533 | 146 | 1064 | 1187 | 1428 | 383 | 2370 | 2932 | 3383 |
| DIC | 112251 | 112209 | 110574 | 110577 | 19284 | 18982 | 18921 | 18920 | 57652 | 57457 | 55275 | 55273 |
| *Controls:* |  |  |  |  |  |  |  |  |  |  |  |  |
| MH hospitals random effects | X | X | X | X | X | X | X | X | X | X | X | X |
| Patient socio-demographic characteristics (set 1) |  | X | X | X |  | X | X | X |  | X | X | X |
| Patient clinical characteristics (set 2) |  |  | X | X |  |  | X | X |  |  | X | X |
| Hospital & LSOA characteristics |  |  |  | X |  |  |  | X |  |  |  | X |

***Notes.*** Patient socio-demographic characteristics (set1): gender, age bands, ethnicity, civil status, LSOA deprivation. Patient clinical characteristics (set2): HONOS and SARN scores, probability of MHCT Best Fit Cluster. Unmatched = Cluster assigned by Clinician is different than Cluster assigned by MHCT algorithm. Non-psychotic & Psychotic patients reference categories are: Male, aged 19-29, white, single, most deprived. Organic patients reference categories are: Male, aged over 80, white, married, most deprived. Burn-in: 5000; Chain: 50000; Thinning: 50; t-statistics in parentheses; * p<0.10, ** p<0.05, *** p<0.01. ‡ Organic Superclass: variable is a binary dummy = 1 if Total SARN score > 0.

## Modelling assignment to specific mental health clusters within a superclass

To explore the differential coding practices between mental health providers, we analyse the probability of a patient being assigned to a specific cluster. Such an analytical step is also important to verify that any results from the main analysis on under-/over-coding is not due to baseline differences in patients’ clustering across providers.

We fit a multinomial logistic model (Hedeker, 2003) with random effects at provider level that is drawn from a multivariate normal distribution, separately for the three superclasses. Formally:

$\Pr\left[ y_{ih}=c \right]=\frac{\exp\left( {X_{ih}}^{'}\beta_{k}+P_{ih}^{'}\delta_{c}+Z_{h}^{'}\theta_{c}+\mu_{hc} \right)}{\sum_{c=K}^{K+L} \exp\left( {X_{ih}}^{'}\beta_{c}+P_{ih}^{'}\delta_{c}+Z_{h}^{'}\theta_{c}+\mu_{hc} \right)}$ (5)

$$\left(\begin{matrix} \mu_{h,K} \\ \mu_{h,K+1} \\ \vdots\\ \mu_{h,K+L} \end{matrix}\right)\sim MVN\left\{\left(\begin{matrix} 0 \\ 0 \\ \vdots\\ 0 \end{matrix}\right),\left(\begin{matrix} \sigma_{K}^{2} & & & \\ \sigma_{K,K+1} & \sigma_{K+1}^{2} & & \\ \vdots& \vdots& \ddots& \\ \sigma_{K,K+L} & \sigma_{K+1,K+L} & \ldots& \sigma_{K+L}^{2} \end{matrix}\right)\right\}$$

where

$$c=\left\{ \begin{matrix} 1,2,3,4,5,6,7,8 & if & Superclass=non-psychotic \\ 10,11,12,13,14,15,16,17 & if & Superclass=psychotic \\ 18,19,20,21 & if & Superclass=organic \end{matrix} \right.$$

and $B\in\left[K,K+L\right]$.

$c$ is the random cluster to which patient *i* in hospital *h* can been assigned among the set $\left[K,...,K+L\right]$, which are the specific clusters for each superclass *s*. $B$is the reference cluster within a superclass, here set to the cluster with the highest number of patients (i.e. most populous) in that superclass. $M$is the number of clusters within a superclass, besides the reference category $B$. $X_{ih}$is a vector of patient-level variables, $Z_{h}$is a vector of provider-specific variables. We compute the MOR for each cluster as $\text{MOR}_{c}=\text{med}\left\{\exp\left[\left(2\sigma_{c}^{2}\right)^{0.5}\times\Phi^{-1}\left(0.75\right)\right]\right\}$, where $\sigma_{c}^{2}$ is the variance of the conditional odds ratio and $c=K,...,K+L$. In our regression analysis, we use the largest clusters 4, 10 and 18 as the reference clusters for the non-psychotic, psychotic and organic superclasses, respectively.

Tables C.I, C.II and C.III show the regression results for this analysis.

After controlling for patient-level case-mix variables and provider-level predictors, a large amount of unexplained provider variation remains. Table C.I presents results for the non-psychotic superclass. The MORs resulting from the provider-level residuals range from 1.82 to 3.59, so that, selecting two hospitals at random, the odds of a given patient being assigned to a specific cluster instead of cluster 4 is between 1.8 and 3.6 times higher than in another provider. Between-provider variation is lower for patients in the psychotic superclass (Table C.II) and mainly concentrated in clusters 13, 16 and 17, so that, when selecting two hospitals at random, the odds of a given patient being assigned to a specific cluster instead of cluster 10 is about 2 times higher than in another provider. Table C.III reports the results for the organic superclass. The estimated MORs for all clusters range between 1.89 and 2.37, and they are always larger than the Odds Ratios of the strongest predictor (a positive Total SARN score).

**Table C.I. Mixed-effects multilevel multinomial logit model – Investigating provider effects in assigning patients to specific clusters in the non-psychotic superclass.**

|  | **1** | **2** | **3** | **4** | **5** | **6** | **7** |
| --- | --- | --- | --- | --- | --- | --- | --- |
|  | **Odds ratio: cluster 1 vs 4** | **Odds ratio: cluster 2 vs 4** | **Odds ratio: cluster 3 vs 4** | **Odds ratio: cluster 5 vs 4** | **Odds ratio: cluster 6 vs 4** | **Odds ratio: cluster 7 vs 4** | **Odds ratio: cluster 8 vs 4** |
| Total HONOS score | 0.7624*** | 0.8398*** | 0.8944*** | 1.0636*** | 0.9788*** | 1.0196*** | 1.0246*** |
|  | (-58.792) | (-49.539) | (-50.247) | (26.340) | (-6.014) | (6.858) | (7.300) |
| Total SARN score‡ | 0.9156*** | 0.9474*** | 0.9752*** | 1.0273*** | 1.2561*** | 1.2145*** | 1.3177*** |
|  | (-10.848) | (-8.714) | (-6.535) | (6.878) | (43.197) | (43.952) | (54.616) |
| Probability of MHCT Best Fit Cluster | 1.0696*** | 1.0499*** | 1.0004 | 1.0353*** | 0.9946** | 0.9938*** | 1.0199*** |
|  | (29.320) | (24.132) | (0.250) | (24.907) | (-2.346) | (-3.232) | (10.920) |
| Num. of hospital patients in 2013/14 (in 1,000) | 0.9889 | 0.9959 | 1.0036 | 0.9845** | 0.9902 | 0.9940 | 0.9997 |
|  | (-0.784) | (-0.323) | (0.399) | (-1.988) | (-1.201) | (-0.646) | (-0.036) |
| % patients - largest commissioning CCG in 2013/14 | 1.0101 | 1.0153* | 1.0116* | 0.9940 | 0.9980 | 0.9930 | 0.9952 |
|  | (1.085) | (1.808) | (1.905) | (-1.266) | (-0.433) | (-1.178) | (-0.909) |
| 1^st^ tercile of average hospital cost per episode in 2013/14 | 0.7304 | 0.7187 | 1.0870 | 0.8607 | 1.0022 | 1.2535 | 1.3384 |
|  | (-0.753) | (-0.915) | (0.372) | (-0.597) | (0.009) | (0.724) | (1.047) |
| 3^rd^ tercile of average hospital cost per episode in 2013/14 | 1.4171 | 1.1130 | 1.2649 | 0.8891 | 0.9744 | 1.3580 | 1.0997 |
|  | (0.825) | (0.291) | (0.872) | (-0.520) | (-0.114) | (1.162) | (0.387) |
| % most deprived quintile patients (hospital-level) | 1.0214 | 1.0210 | 0.9975 | 1.0031 | 0.9979 | 0.9877 | 1.0110 |
|  | (0.711) | (0.781) | (-0.147) | (0.270) | (-0.177) | (-0.898) | (0.850) |
| Staff Engagement (% agree or more) | 0.8968** | 0.9132** | 0.9655 | 1.0233 | 1.0077 | 1.0096 | 1.0132 |
|  | (-2.369) | (-2.265) | (-1.169) | (0.875) | (0.303) | (0.281) | (0.414) |
| ICC (Intraclass Correlation Coefficient) | 0.3393 | 0.2860 | 0.1609 | 0.1143 | 0.1091 | 0.1573 | 0.1357 |
| MOR (Median Odds Ratio) | 3.4550 | 2.9894 | 2.1334 | 1.8620 | 1.8319 | 2.1117 | 1.9846 |
| MOR 95% Cred. Int. L. Bound | 2.6976 | 2.3726 | 1.8436 | 1.6586 | 1.6311 | 1.8424 | 1.7353 |
| MOR 95% Cred. Int. U. Bound | 4.7419 | 3.9114 | 2.5460 | 2.1701 | 2.1254 | 2.5315 | 2.3212 |
| Probability (unmatched assignment) | 0.0146 | 0.0439 | 0.2076 | 0.1511 | 0.0756 | 0.0719 | 0.0384 |
| Prob. (unmatched assignment) 95% Cred. Int. L. Bound | 0.0080 | 0.0270 | 0.1591 | 0.1149 | 0.0569 | 0.0536 | 0.0278 |
| Prob. (unmatched assignment) 95% Cred. Int. U. Bound | 0.0256 | 0.0704 | 0.2628 | 0.1957 | 0.0969 | 0.0965 | 0.0513 |

***Notes****.* Number of patients: 87,265; Computational Time (in seconds): 179,231; DIC: 271,773. Reference categories are: Male, aged 19-29, white, single, most deprived. The effects of some patient-level variables (age bands indicators, gender, deprivation quintiles, marital status, ethnicity) are not reported here but are available from the authors. Burn-in: 5000; Chain: 50,000; Thinning: 50; t-statistics in parentheses; * p<0.10, ** p<0.05, *** p<0.01. Estimated probability [95% Confidence Interval bounds] of clinician assignment to baseline MH cluster 4 = 0.3785 [0.3215; 0.4313]. ^‡^ Organic Superclass: variable is a binary dummy = 1 if Total SARN score > 0.

|  | **1** | **2** | **3** | **4** | **5** | **6** | **7** |
| --- | --- | --- | --- | --- | --- | --- | --- |
|  | **Odds ratio: cluster 11 vs 10** | **Odds ratio: cluster 12 vs 10** | **Odds ratio: cluster 13 vs 10** | **Odds ratio: cluster 14 vs 10** | **Odds ratio: cluster 15 vs 10** | **Odds ratio: cluster 16 vs 10** | **Odds ratio: cluster 17 vs 10** |
| Total HONOS score | 0.8879*** | 0.9755*** | 1.0305*** | 1.0372*** | 1.0923*** | 1.0597*** | 0.9946 |
|  | (-24.625) | (-5.004) | (5.506) | (8.386) | (14.192) | (8.293) | (-0.655) |
| Total SARN score^‡^ | 1.0774*** | 1.1179*** | 1.1136*** | 1.0662*** | 0.9644*** | 1.0739*** | 1.2565*** |
|  | (9.650) | (13.669) | (12.013) | (8.928) | (-3.377) | (6.118) | (16.776) |
| Probability of MHCT Best Fit Cluster | 1.0393*** | 0.9398*** | 0.9588*** | 1.0395*** | 1.1707*** | 1.1586*** | 1.0820*** |
|  | (2.737) | (-3.861) | (-2.648) | (3.350) | (11.130) | (10.427) | (4.577) |
| Num. of hospital patients in 2013/14 (in 1,000) | 0.9976 | 1.0093 | 1.0133 | 0.9927 | 0.9963 | 0.9965 | 1.0002 |
|  | (-0.403) | (1.145) | (1.215) | (-0.946) | (-0.419) | (-0.302) | (0.019) |
| % patients - largest commissioning CCG in 2013/14 | 1.0006 | 0.9968 | 0.9929 | 0.9928* | 0.9933 | 0.9940 | 0.9974 |
|  | (0.175) | (-0.725) | (-1.163) | (-1.651) | (-1.360) | (-0.838) | (-0.414) |
| 1^st^ tercile of average hospital cost per episode in 2013/14 | 0.8975 | 1.2841 | 1.2249 | 0.9917 | 1.3358 | 1.3378 | 1.1382 |
|  | (-0.687) | (1.245) | (0.754) | (-0.042) | (1.343) | (0.992) | (0.498) |
| 3^rd^ tercile of average hospital cost per episode in 2013/14 | 0.6040*** | 0.7672 | 0.7628 | 0.8151 | 1.2202 | 0.6328 | 0.8783 |
|  | (-3.087) | (-1.243) | (-0.962) | (-1.101) | (0.861) | (-1.532) | (-0.503) |
| % most deprived quintile patients (hospital-level) | 0.9993 | 0.9952 | 0.9941 | 0.9859 | 0.9959 | 0.9849 | 1.0069 |
|  | (-0.093) | (-0.494) | (-0.438) | (-1.508) | (-0.383) | (-1.006) | (0.515) |
| Staff Engagement (% agree or more) | 0.9919 | 1.0085 | 0.9998 | 1.0207 | 0.9607* | 0.9746 | 0.9989 |
|  | (-0.463) | (0.393) | (-0.008) | (0.961) | (-1.733) | (-0.755) | (-0.036) |
| ICC (Intraclass Correlation Coefficient) | 0.0462 | 0.0723 | 0.1302 | 0.0717 | 0.0813 | 0.1578 | 0.1098 |
| MOR (Median Odds Ratio) | 1.4637 | 1.6209 | 1.9529 | 1.6174 | 1.6733 | 2.1149 | 1.8360 |
| MOR 95% Cred. Int. L. Bound | 1.3409 | 1.4610 | 1.6888 | 1.4494 | 1.4683 | 1.7906 | 1.5615 |
| MOR 95% Cred. Int. U. Bound | 1.6416 | 1.8289 | 2.3171 | 1.8452 | 1.9412 | 2.5585 | 2.2164 |
| Probability (cluster = J) | 0.0947 | 0.0589 | 0.0427 | 0.1480 | 0.0165 | 0.0370 | 0.0096 |
| Prob. (clust.=J) 95% Cred. Int. L. Bound | 0.0732 | 0.0433 | 0.0275 | 0.1118 | 0.0108 | 0.0213 | 0.0053 |
| Prob. (clust.=J) 95% Cred. Int. U. Bound | 0.1203 | 0.0767 | 0.0622 | 0.1896 | 0.0235 | 0.0602 | 0.0154 |

**Table C.II. Mixed-effects multilevel multinomial logit model – Investigating provider effects in assigning patients to specific clusters in the psychotic superclass.**

***Notes****.* Number of patients: 16,941; Computational Time (in seconds): 33,270; DIC: 54,204. Reference categories are: Male, aged 19-29, white, single, most deprived. The effects of some patient-level variables (age bands indicators, gender, deprivation quintiles, marital status, ethnicity) are not reported here but are available from the authors. Burn-in: 5,000; Chain: 50,000; Thinning: 50; t-statistics in parentheses; * p<0.10, ** p<0.05, *** p<0.01. Estimated probability [95% Confidence Interval bounds] of clinician assignment to baseline MH cluster 10 = 0.5839 [0.5321; 0.6356]. ^‡^ Organic Superclass: variable is a binary dummy = 1 if Total SARN score > 0.

**Table C.III. Mixed-effects multilevel multinomial logit model – Investigating provider effects in assigning patients to specific clusters in the organic superclass.**

|  | **1** | **2** | **3** |
| --- | --- | --- | --- |
|  | **Odds ratio: cluster 19 vs 18** | **Odds ratio: cluster 20 vs 18** | **Odds ratio: cluster 21 vs 18** |
| Total HONOS score | 1.2590*** | 1.4615*** | 1.4987*** |
|  | (73.341) | (80.185) | (63.118) |
| Total SARN score^‡^ | 1.4613*** | 2.1000*** | 1.7933*** |
|  | (13.913) | (15.607) | (8.035) |
| Probability of MHCT Best Fit Cluster | 0.9617*** | 0.9926*** | 1.0194*** |
|  | (-18.390) | (-3.296) | (7.780) |
| Num. of hospital patients in 2013/14 (in 1,000) | 1.0051 | 0.9933 | 1.0036 |
|  | (0.607) | (-0.636) | (0.268) |
| % patients - largest commissioning CCG in 2013/14 | 1.0004 | 0.9980 | 1.0021 |
|  | (0.071) | (-0.295) | (0.261) |
| 1^st^ tercile of average hospital cost per episode in 2013/14 | 1.3139 | 0.8617 | 1.0080 |
|  | (1.060) | (-0.483) | (0.022) |
| 3^rd^ tercile of average hospital cost per episode in 2013/14 | 1.3667 | 1.1199 | 1.3679 |
|  | (1.258) | (0.366) | (0.839) |
| % most deprived quintile patients (hospital-level) | 1.0095 | 1.0309* | 1.0294 |
|  | (0.705) | (1.863) | (1.502) |
| Staff Engagement (% agree or more) | 0.9803 | 0.9788 | 0.9835 |
|  | (-0.710) | (-0.638) | (-0.426) |
| ICC (Intraclass Correlation Coefficient) | 0.1208 | 0.1663 | 0.2160 |
| MOR (Median Odds Ratio) | 1.8987 | 2.1655 | 2.4797 |
| MOR 95% Cred. Int. L. Bound | 1.6674 | 1.8555 | 2.0851 |
| MOR 95% Cred. Int. U. Bound | 2.2252 | 2.6012 | 3.0864 |
| Probability (cluster = J) | 0.3538 | 0.0401 | 0.0097 |
| Prob. (clust.=J) 95% Cred. Int. L. Bound | 0.2791 | 0.0278 | 0.0058 |
| Prob. (clust.=J) 95% Cred. Int. U. Bound | 0.4386 | 0.0564 | 0.0151 |

***Notes:*** Number of patients: 44,266; Computational Time (in seconds): 17,656; DIC: 72,895. Reference categories are: Male, aged over 80, white, married, most deprived. The effects of some patient-level variables (age bands indicators, gender, deprivation quintiles, marital status, ethnicity) are not reported here but are available from the authors. Burn-in: 5,000; Chain: 50,000; Thinning: 50; t-statistics in parentheses; * p<0.10, ** p<0.05, *** p<0.01. Estimated probability [95% Confidence Interval bounds] of clinician assignment to baseline MH cluster 18 = 0.5862 [0.4975; 0.6832]. ^‡^ Organic Superclass: variable is a binary dummy = 1 if Total SARN score > 0).

## Robustness check: determinants of mismatch, using 50% random draws from the full sample

|  | **Mixed-effects Binary Logit Model** | | |  | **Mixed-effects Multinomial Logit Model** | | | | | |
| --- | --- | --- | --- | --- | --- | --- | --- | --- | --- | --- |
|  | **Non-psychotic** | **Psychotic** | **Organic** |  | **Non-Psychotic** | | **Psychotic** | | **Organic** | |
| **Odds Ratio** | **1** | **2** | **3** |  | **4** | **5** | **6** | **7** | **8** | **9** |
|  | **Odds ratio: unmatched VS matched** | **Odds ratio: unmatched VS matched** | **Odds ratio: unmatched VS matched** |  | **Odd ratio: *underclustered* VS matched** | **Odd ratio: *overclustered* VS matched** | **Odd ratio: *underclustered* VS matched** | **Odd ratio: *overclustered* VS matched** | **Odd ratio: *underclustered* VS matched** | **Odd ratio: *overclustered* VS matched** |
| Total HONOS score | 0.9758*** | 1.0313*** | 1.0519*** |  | 0.9871*** | 0.9638*** | 1.0405*** | 1.0116*** | 1.0815*** | 1.0229*** |
|  | (-23.969) | (27.062) | (33.697) |  | (-10.932) | (-31.110) | (31.578) | (7.608) | (41.938) | (11.995) |
| Total SARN score ‡ | 1.0431*** | 1.0330*** | 1.1769*** |  | 1.0929*** | 0.9946*** | 1.0429*** | 0.9971 | 1.2913*** | 1.0859*** |
|  | (26.585) | (16.903) | (8.851) |  | (47.940) | (-2.912) | (19.803) | (-1.134) | (10.549) | (3.668) |
| Probability of MHCT Best Fit Cluster | 0.9760*** | 0.9800*** | 0.9848*** |  | 0.9744*** | 0.9764*** | 1.0132*** | 0.8087*** | 0.9976*** | 0.9564*** |
|  | (-45.146) | (-8.570) | (-20.806) |  | (-40.146) | (-34.684) | (5.073) | (-38.844) | (-2.810) | (-35.433) |
| Number of hospital patients in 2013/14 | 0.9923* | 0.9918** | 0.9899** |  | 0.9879* | 0.9907* | 0.9883** | 0.9958 | 0.9829*** | 0.9952 |
|  | (-1.665) | (-2.228) | (-2.353) |  | (-1.874) | (-1.702) | (-2.493) | (-0.873) | (-2.920) | (-1.043) |
| % patients from largest CCG in 2013/14 | 1.0021 | 0.9979 | 1.0017 |  | 1.0025 | 0.9984 | 0.9971 | 0.9964 | 0.9962 | 1.0047* |
|  | (0.721) | (-0.931) | (0.727) |  | (0.679) | (-0.524) | (-1.101) | (-1.314) | (-0.993) | (1.649) |
| 1st tercile of average cost per episode in 2013/14 | 0.8046 | 0.8672 | 0.8110* |  | 0.7141* | 0.8715 | 0.8181 | 0.9324 | 0.8440 | 0.7786** |
|  | (-1.488) | (-1.282) | (-1.919) |  | (-1.869) | (-0.890) | (-1.619) | (-0.533) | (-0.967) | (-2.054) |
| 3rd tercile of average cost per episode in 2013/14 | 0.8900 | 0.9899 | 0.9248 |  | 0.8364 | 0.9609 | 0.9312 | 1.0407 | 0.8596 | 1.0556 |
|  | (-0.881) | (-0.089) | (-0.669) |  | (-1.022) | (-0.232) | (-0.535) | (0.320) | (-0.866) | (0.439) |
| % most deprived quintile patients (hospital-level) | 1.0208*** | 1.0059 | 1.0129** |  | 1.0166* | 1.0170** | 1.0076 | 1.0027 | 1.0084 | 1.0175*** |
|  | (2.624) | (0.989) | (2.164) |  | (1.888) | (2.442) | (1.180) | (0.428) | (0.941) | (2.746) |
| Staff Engagement (% agree or more) | 0.9974 | 0.9967 | 0.9878 |  | 0.9721** | 1.0197 | 0.9891 | 1.0108 | 0.9741 | 1.0011 |
|  | (-0.165) | (-0.253) | (-1.043) |  | (-2.101) | (1.185) | (-0.850) | (0.782) | (-1.165) | (0.081) |
| ICC (Intraclass Correlation Coefficient) | 0.0448 | 0.0319 | 0.0298 |  | 0.0667 | 0.0560 | 0.0388 | 0.0412 | 0.0643 | 0.0336 |
| MOR (Median Odds Ratio) | 1.4545 | 1.3686 | 1.3543 |  | 1.5878 | 1.5240 | 1.4160 | 1.4312 | 1.5741 | 1.3810 |
| MOR 95% Cred. Int. L. Bound | 1.3579 | 1.2877 | 1.2727 |  | 1.4516 | 1.4050 | 1.3250 | 1.3378 | 1.4408 | 1.2929 |
| MOR 95% Cred. Int. U. Bound | 1.5941 | 1.4700 | 1.4528 |  | 1.7603 | 1.6785 | 1.5407 | 1.5530 | 1.7619 | 1.4986 |
| Probability (*unmatched* assignment) | 0.6808 | 0.7727 | 0.4260 |  | 0.3252 | 0.3421 | 0.6078 | 0.1624 | 0.1877 | 0.2333 |
| Prob. (*unmatched* assignment) 95% Cred. Int. L. Bound | 0.6342 | 0.7411 | 0.3853 |  | 0.2787 | 0.2944 | 0.5654 | 0.1387 | 0.1490 | 0.2020 |
| Prob. (*unmatched* assignment) 95% Cred. Int. U. Bound | 0.7185 | 0.8018 | 0.4688 |  | 0.3796 | 0.3957 | 0.6464 | 0.1872 | 0.2287 | 0.2650 |
| Observations | 164,785 | 96,188 | 79,036 |  | 164,785 | | 96,188 | | 79,036 | |
| Time | 14,233 | 8,287 | 6,127 |  | 46,664 | | 26,741 | | 19,153 | |
| DIC | 206,652 | 120,014 | 105,086 |  | 340,428 | | 190,845 | | 148,799 | |

**Table D.I. Mismatch between clinician assignment and MHCT algorithm assignment. Mixed-effects multilevel multinomial logit model - 50% random draws from the full sample (to be compared with Table V in the main manuscript).**

***Notes****.* Columns 1-3: Unmatched = Cluster assigned by Clinician is different than Cluster assigned by MHCT algorithm. Columns 4-9: Unmatched = Cluster assigned by Clinician is higher (*overclustered*) or lower (*underclustered*) than Cluster assigned by MHCT algorithm. Non-psychotic & Psychotic patients reference categories are: Male, aged 19-29, white, single, most deprived. Organic patients reference categories are: Male, aged over 80, white, married, most deprived. Burn-in: 5,000; Chain: 50,000; Thinning: 50; t-statistics in parentheses; * p<0.10, ** p<0.05, *** p<0.01. ^‡^ Organic Superclass: variable is a binary dummy = 1 if Total SARN score > 0.

|  | **Mixed-effects Binary Logit Model** | | | | |  | **Mixed-effects Multinomial Logit Model** | | | | | | |
| --- | --- | --- | --- | --- | --- | --- | --- | --- | --- | --- | --- | --- | --- |
|  | **Non-psychotic** | **Psychotic** | **Organic** |  | **Non-Psychotic** | | | | **Psychotic** | | **Organic** | |  |
| **Odds Ratio** | **1** | **2** | **3** |  | **4** | | | **5** | **6** | **7** | **8** | **9** |  |
|  | **Odds ratio: unmatched VS matched** | **Odds ratio: unmatched VS matched** | **Odds ratio: unmatched VS matched** |  | **Odd ratio: *underclustered* VS matched** | | | **Odd ratio: *overclustered* VS matched** | **Odd ratio: *underclustered* VS matched** | **Odd ratio: *overclustered* VS matched** | **Odd ratio: *underclustered* VS matched** | **Odd ratio: *overclustered* VS matched** |  |
| Total HONOS score | 0.9758*** | 1.0313*** | 1.0519*** |  | 0.9867*** | | | 0.9645*** | 1.0483*** | 1.0130*** | 1.0815*** | 1.0229*** |  |
|  | (-23.969) | (27.062) | (33.697) |  | (-11.197) | | | (-30.701) | (36.274) | (9.384) | (41.938) | (11.995) |  |
| Total SARN score ‡ | 1.0431*** | 1.0330*** | 1.1769*** |  | 1.0870*** | | | 0.9972 | 1.0725*** | 0.9899*** | 1.2913*** | 1.0859*** |  |
|  | (26.585) | (16.903) | (8.851) |  | (45.386) | | | (-1.523) | (31.652) | (-4.425) | (10.549) | (3.668) |  |
| Probability of MHCT Best Fit Cluster | 0.9760*** | 0.9800*** | 0.9848*** |  | 0.9767*** | | | 0.9734*** | 0.9946** | 0.9264*** | 0.9976*** | 0.9564*** |  |
|  | (-45.146) | (-8.570) | (-20.806) |  | (-36.820) | | | (-39.263) | (-2.081) | (-21.312) | (-2.810) | (-35.433) |  |
| Number of hospital patients in 2013/14 | 0.9923* | 0.9918** | 0.9899** |  | 0.9912* | | | 0.9950 | 0.9853*** | 0.9962 | 0.9829*** | 0.9952 |  |
|  | (-1.665) | (-2.228) | (-2.353) |  | (-1.647) | | | (-1.102) | (-2.909) | (-0.733) | (-2.920) | (-1.043) |  |
| % patients from largest CCG in 2013/14 | 1.0021 | 0.9979 | 1.0017 |  | 1.0026 | | | 0.9998 | 0.9975 | 0.9970 | 0.9962 | 1.0047* |  |
|  | (0.721) | (-0.931) | (0.727) |  | (0.724) | | | (-0.070) | (-0.837) | (-0.982) | (-0.993) | (1.649) |  |
| 1st tercile of average cost per episode in 2013/14 | 0.8046 | 0.8672 | 0.8110* |  | 0.7119* | | | 0.8535 | 0.7818* | 0.8176 | 0.8440 | 0.7786** |  |
|  | (-1.488) | (-1.282) | (-1.919) |  | (-1.888) | | | (-1.050) | (-1.689) | (-1.333) | (-0.967) | (-2.054) |  |
| 3rd tercile of average cost per episode in 2013/14 | 0.8900 | 0.9899 | 0.9248 |  | 0.8164 | | | 0.9428 | 0.9346 | 1.0479 | 0.8596 | 1.0556 |  |
|  | (-0.881) | (-0.089) | (-0.669) |  | (-1.164) | | | (-0.370) | (-0.512) | (0.359) | (-0.866) | (0.439) |  |
| % most deprived quintile patients (hospital-level) | 1.0208*** | 1.0059 | 1.0129** |  | 1.0155* | | | 1.0156** | 1.0095 | 1.0055 | 1.0084 | 1.0175*** |  |
|  | (2.624) | (0.989) | (2.164) |  | (1.753) | | | (2.231) | (1.556) | (0.929) | (0.941) | (2.746) |  |
| Staff Engagement (% agree or more) | 0.9974 | 0.9967 | 0.9878 |  | 0.9795 | | | 1.0277 | 0.9835 | 1.0145 | 0.9741 | 1.0011 |  |
|  | (-0.165) | (-0.253) | (-1.043) |  | (-1.088) | | | (1.616) | (-0.999) | (0.905) | (-1.165) | (0.081) |  |
| ICC (Intraclass Correlation Coefficient) | 0.0448 | 0.0319 | 0.0298 |  | 0.0723 | | | 0.0521 | 0.0417 | 0.0414 | 0.0643 | 0.0336 |  |
| MOR (Median Odds Ratio) | 1.4545 | 1.3686 | 1.3543 |  | 1.6209 | | | 1.5001 | 1.4348 | 1.4324 | 1.5741 | 1.3810 |  |
| MOR 95% Cred. Int. L. Bound | 1.3579 | 1.2877 | 1.2727 |  | 1.4798 | | | 1.3931 | 1.3264 | 1.3339 | 1.4408 | 1.2929 |  |
| MOR 95% Cred. Int. U. Bound | 1.5941 | 1.4700 | 1.4528 |  | 1.8202 | | | 1.6501 | 1.5587 | 1.5578 | 1.7619 | 1.4986 |  |
| Probability (*unmatched* assignment) | 0.6808 | 0.7727 | 0.4260 |  | 0.3214 | | | 0.3510 | 0.3199 | 0.4557 | 0.1877 | 0.2333 |  |
| Prob. (*unmatched* assignment) 95% Cred. Int. L. Bound | 0.6342 | 0.7411 | 0.3853 |  | 0.2747 | | | 0.3044 | 0.2827 | 0.4171 | 0.1490 | 0.2020 |  |
| Prob. (*unmatched* assignment) 95% Cred. Int. U. Bound | 0.7185 | 0.8018 | 0.4688 |  | 0.3780 | | | 0.3991 | 0.3561 | 0.4986 | 0.2287 | 0.2650 |  |
| Observations | 164785 | 96188 | 79036 |  | 164785 | | | | 96188 | | 79036 | |  |
| Time | 14233 | 8287 | 6127 |  | 42378 | | | | 24446 | | 19153 | |  |
| DIC | 206652 | 120014 | 105086 |  | 340418 | | | | 196867 | | 148799 | |  |

**Table D.II. Mismatch between clinician assignment and MHCT algorithm assignment, with clusters order on average costs per episode. Mixed-effects multilevel multinomial logit model – 50% random draws from the full sample (to be compared with Table VI in the main manuscript).**

***Notes****.* Columns 1-3: Unmatched = Cluster assigned by Clinician is different than Cluster assigned by MHCT algorithm. Columns 4-9: Unmatched = Cluster assigned by Clinician is higher (*overclustered*) or lower (*underclustered*) than Cluster assigned by MHCT algorithm. Non-psychotic & Psychotic patients reference categories are: Male, aged 19-29, white, single, most deprived. Organic patients reference categories are: Male, aged over 80, white, married, most deprived. Burn-in: 5,000; Chain: 50,000; Thinning: 50; t-statistics in parentheses; * p<0.10, ** p<0.05, *** p<0.01. ^‡^ Organic Superclass: variable is a binary dummy = 1 if Total SARN score > 0.
